# A Highly Efficient Sensor Platform Using Simply Manufactured Nanodot Patterned Substrates

**DOI:** 10.1038/srep13270

**Published:** 2015-08-20

**Authors:** Sozaraj Rasappa, Tandra Ghoshal, Dipu Borah, Ramsankar Senthamaraikannan, Justin D. Holmes, Michael A. Morris

**Affiliations:** 1 Materials Research Group, Department of Chemistry and Tyndall National Institute, University College Cork, Cork, Ireland; 2 Centre for Research on Adaptive Nanostructures and Nanodevices (CRANN), Trinity College Dublin, Dublin, Ireland; 3 AMBER (Advanced Material and Bio-Engineering Research Centre), Trinity College Dublin, Dublin-2, Ireland

## Abstract

Block copolymer (BCP) self-assembly is a low-cost means to nanopattern surfaces. Here, we use these nanopatterns to directly print arrays of nanodots onto a conducting substrate (Indium Tin Oxide (ITO) coated glass) for application as an electrochemical sensor for ethanol (EtOH) and hydrogen peroxide (H_2_O_2_) detection. The work demonstrates that BCP systems can be used as a highly efficient, flexible methodology for creating functional surfaces of materials. Highly dense iron oxide nanodots arrays that mimicked the original BCP pattern were prepared by an ‘insitu’ BCP inclusion methodology using poly(styrene)-*block*-poly(ethylene oxide) (PS-*b*-PEO). The electrochemical behaviour of these densely packed arrays of iron oxide nanodots fabricated by two different molecular weight PS-*b*-PEO systems was studied. The dual detection of EtOH and H_2_O_2_ was clearly observed. The as-prepared nanodots have good long term thermal and chemical stability at the substrate and demonstrate promising electrocatalytic performance.

Electrochemical sensors offer elegant routes for interfacing, at the molecular level, chemical or biological recognition events and electronic signal-transduction processes for meeting the size, cost, low-volume and power requirements of decentralized testing and are highly promising candidates in a wide range of biomedical or environmental applications^1^,^2^,^3^,^4^,^5^. However, the low-cost fabrication of highly efficient electrochemical nanoengineered sensors of high reproducibility and stability is a challenge^6^. Here, we propose a simple methodology for nanoengineering electrodes surfaces that could be a general technique for making a number of different sensors.

Fast and reliable determination of hydrogen peroxide and ethanol (EtOH) in the food, pharmaceutical, clinical, industrial and environmental industry has prompted extensive investigation of various forms of electrochemical sensor^7^,^8^,^9^ and these two chemicals are suitable choices to demonstrate the methodology outlined here. Metal/metal oxide nanoparticles and nanocomposites immobilized on a working electrode surface have attracted substantial interest as sensing elements because of their high surface area and can be formed via a range of methods including physical adsorption, chemical covalent bonding, electrodeposition, electropolymerization and so on^10^,^11^,^12^. Magnetic nanoparticles (such as Fe_3_O_4_) coated with metal, enzyme functionalized or even bare have been used to detect these compounds separately^13^,^14^,^15^. However, simultaneous detection of H_2_O_2_ and EtOH is problematical^16^. Further, whilst nanoparticle based sensors can show good initial activity, accumulation effects can lead to loss of the sensor material surface area and deactivation of the sensor^17^. Thus, development of nanoengineered surfaces of high surface area and physical and chemical stability is required for practical use in the electrochemical sensing area.

ITO coated glass is a common substrate for electrochemical sensor applications being electrically conductive and low cost. However patterning (i.e. generating surface topography to deliver high surface area and hence sensitivity) is a challenge. Precise nanopatterned substrates can be prepared using various lithographic approaches including UV-light, electron beam, optical interference, x-rays or nanoimprint^17^,^18^ but are limited by high cost and low throughput and have had limited impact. BCP self-assembly may be the basis of an alternative, low cost technique for surface nanoengineering allowing both structural and dimension control^19^,^20^,^21^. Here, we have used our established methodology for silicon substrates^22^ to produce ordered iron oxide nanopatterns on ITO. These well-defined arrays were used for the electrochemical sensing of EtOH and H_2_O_2_ and their performance quantified in terms of their density, stability and sensitivity. To the best of our knowledge, this is the first demonstration of this methodology for the single and dual detection of EtOH and H_2_O_2_ which illustrates the capability of these technique.

## Results

### Iron Oxide Nanodot Fabrication from PS-*b*-PEO Films

**Figure 1:**
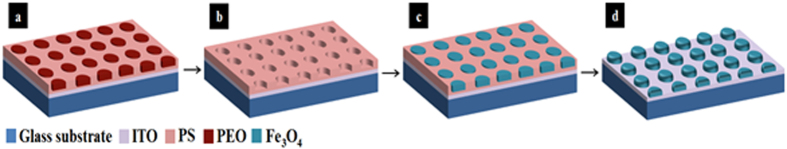
Schematic of Fe_3_O_4_ nanodots fabrication. (**a**) Deposition of PS-*b*-PEO on the ITO glass substrate. (**b**) Wet etch removal of PEO and formation of a porous template. (**c**) Deposition of iron nitrate solution on the PS template. (**d**) Formation of Fe_3_O_4_ dots after PS removal using UV/Ozone.

**Figure 2:**
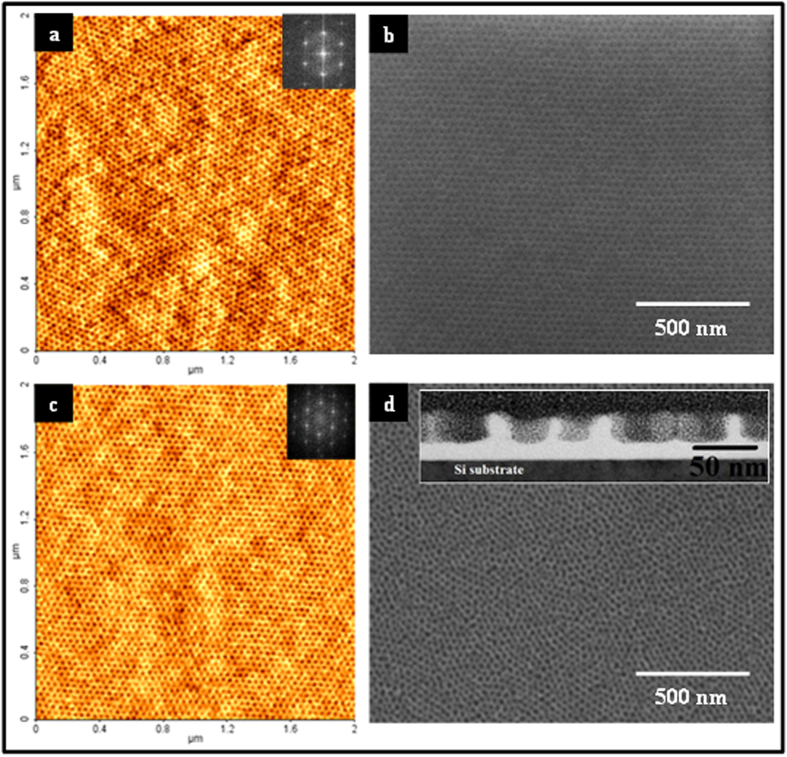
AFM (**a**,**c**) and SEM images (**b**,**d**) of BCP template films. ALW data shown in (**a**,**b**) whilst BHW data in (**c**,**d**). Insets of (**a**,**c**) show the FFT patterns of the corresponding images. (**b**,**d**) templates of ALW and BLW respectively after ethanol treatment. Inset of (**d**) cross-sectional TEM image of BLW after ethanol treatment.

As-coated BCP films exhibit little sign of ordered microphase separated patterns prior to solvent annealing. Figure 1 schematically illustrates the solvent annealing of the films resulted in formation of ordered arrangements of hexagonally packed PEO cylinders (vertical to the surface plane) within a PS matrix. Figure 2a,c show representative tapping mode AFM images of sample ALW and BHW high and low molecular weight samples (see below) after solvent annealing. The FFT (Fourier filtered) patterns shown in the insets of the Fig. 2a,c confirm the periodic nature of the BCP patterns. The films are of regular thicknesses of 25 nm (ALW) and 40 nm (BHW) with little signs of de-wetting and are well-ordered across the entire substrate. The corresponding measured average cylinder centre to centre distances and PEO cylinder diameters for ALW are 32 and 17 nm respectively and the corresponding values for BHW are 42 and 19.3 nm.

In order to fabricate the highest quality nanodot patterns it was necessary to remove/modify the PEO component by ethanolic etch. Figure 2b,d show the SEM images of the PS-*b*-PEO systems after the optimized ultrasonic ethanol treatment for 15 min and 17 min for sample ALW and BHW, respectively. Note that longer exposure to ethanol and higher temperature resulted in surface roughness or structural degradation of the film. Note that this etch treatment allows the SEM imaging reported here due to increased compositional contrast as the PEO was removed. No thickness change (ellipsometry) or deformation was observed following this ethanolic treatment.

The removal of the majority of PEO by ethanol treatment was confirmed by FIB-thinned cross-sectional TEM data of sample BHW and shown in the inset of Fig. 2d. The TEM derived thickness of the film is consistent with ellipsometery measurements. The measured diameters and depths of the PEO-derived regions were 20 nm (in agreement with SEM and AFM) and 28 nm, respectively. No deformation or detachment of the film could be observed. Thus, it can be concluded that the ethanol ultrasonic treatment results a very smooth film surface and the data suggests that it only affects the PEO component.

**Figure 3:**
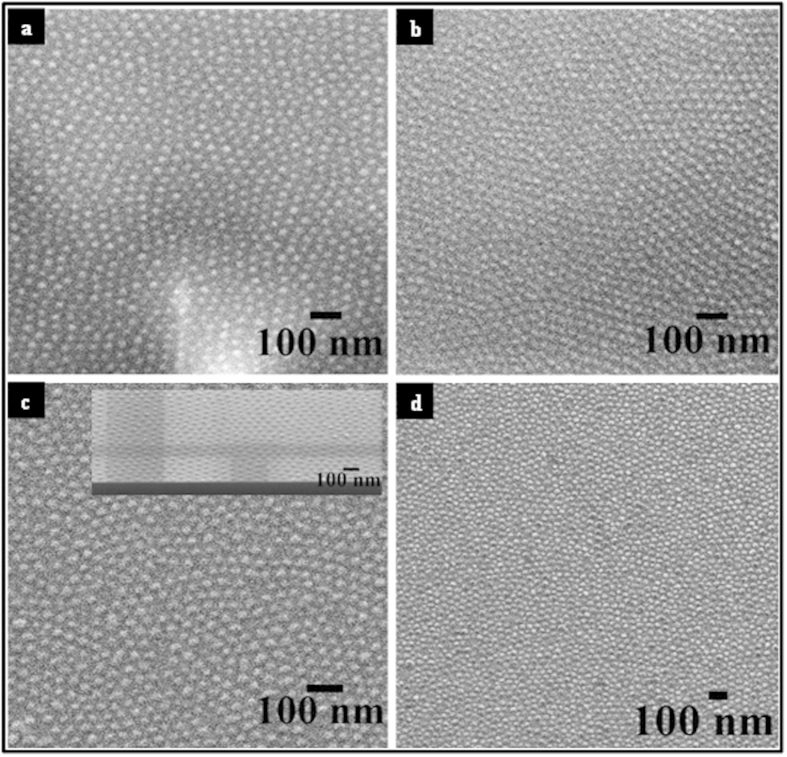
Top-down SEM images of iron oxide nanodots before (**a**,**c**) and after electrochemical (**b**,**d**) analysis. Data from ALW are shown in (**a**,**b**) and corresponding data for BHW in (**c**,**d**) respectively.

The selective inclusion of iron into the nanoporous template is a direct result of the hydrophobic nature of PS. Figure 3a,c show SEM images of well-ordered iron oxide nanodots arrays formed after precursor inclusion followed by UV/Ozone treatment for ALW and BHW. The nanodots have uniform size, shape and their placement mimics the original self-assembled BCP patterns. The average diameters of the nanodots were 18 (ALW) and 24 nm (BLW) when a 0.4 wt% iron precursor solution was used. The average heights of the nanodots were in the range of 6–9 nm as measured by ellipsometry. The density of the nanodots on the substrate is measured approximately 1.1 × 10^11^ and 4.2 × 10^10^ nanodots cm^−2^ for ALW and BHW respectively. The stability and attachment of the nanodots were examined after repeated electrochemical testing in solutions containing H_2_O_2_ and EtOH for 2 h. Figure 3b,d show SEM images following use indicate little, if any, change in the nanodot arrangements and indicate the robustness of these samples in this application. Further evidence of this robustness is detailed below.

**Figure 4:**
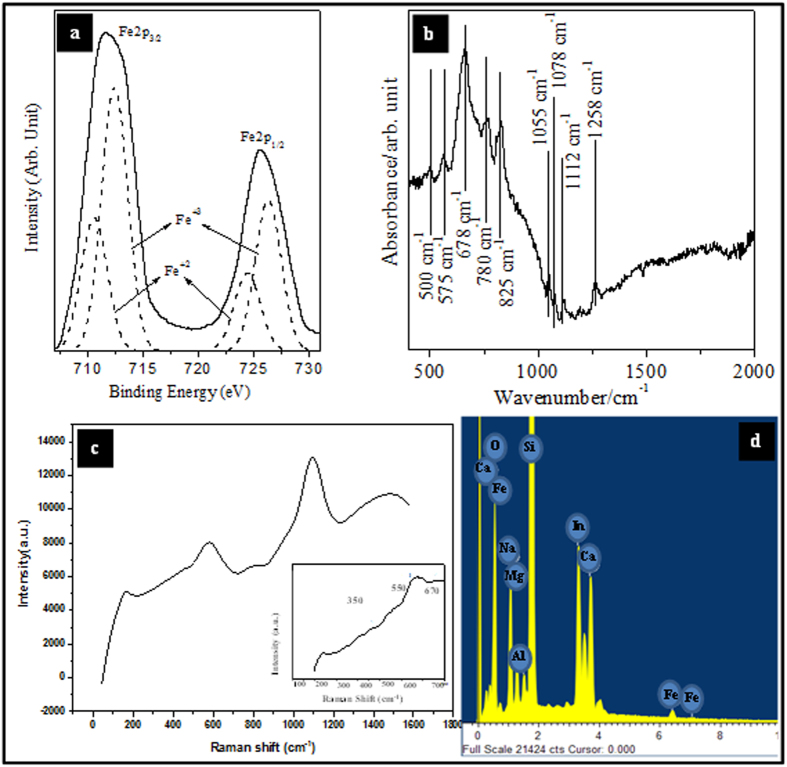
Spectroscopic characterization of Fe_3_O_4_ dots. (**a**) XPS data, (**b**) ATR-FTIR, (**c**) Raman analysis and (**d**) EDX data. See test for details.

XPS was used to confirm the crystalline phase and surface composition of the as-formed nanodots. Figure 4a shows high resolution Fe2p core level spectra (pass energy = 20 eV) of iron oxide nanodots prepared after UV/Ozone treatment. The data consists of two peaks associated with signals due to Fe 2p_3/2_ at 711 eV and Fe 2p_1/2_ at 724.4 eV. Both features are broadened due to the existence of both Fe^2+^ and Fe^3+^ ions^22^,^23^. The Fe 2p_3/2_ and Fe 2p_1/2_ binding energies (BEs) for Fe^2+^ and Fe^3+^ were determined by curve-fitting using Gaussian-Lorentzian line shapes. The measured Fe 2p_3/2_ and Fe 2p_1/2_ BEs are 709.7 and 723 eV (assigned to Fe^2+^) and 711.6 and 725 eV (Fe^3+^) are consistent with literature values for these species^22^,^23^. The ratio of Fe^3+^/Fe^2+^ was calculated from the curve-fitted peak areas as about 2:1 as expected for Fe_3_O_4_ and it is suggested this is the predominant phase present.

Figure 4b shows ATR-FTIR absorption spectra of Fe_3_O_4_ nanodots (from ALW) on ITO to further verify the composition (similar data were recorded for BHW materials). The spectral envelope is consistent with previous data recorded from glass substrates^23^. The features at 1055, 1078 and 1258 cm^−1^ are associated with stretching vibration modes of the Si-O-Si bonds in silicon dioxide^24^. The peak at 1112 cm^−1^ confirms the presence of sulphate contaminants in the glass^25^. The band centred at 678 cm^−1^ can be assigned to neutral charged oxygen vacancies possibly non-bridging oxygen hole centres (NBOHCs) or similar compounds in β-cristobalite, a silica polymorph^26^. The small absorption peaks at 500 and 780 cm^−1^ are associated with In-O and Sn-O bonds^27^. An intense peak at 575 cm^−1^ is due to the most intense peak for magnetite^28^ and thus the data are consistent with the XPS analysis. Raman spectrum (Fig. 4c) provides clear confirmation of the iron oxide phase. Three peaks at 350, 550 and 670 cm^−1^ can be attributed to T_2g.3_, T_2g.2_ and A_1g_ vibrational modes of Fe_3_O_4_, respectively whilst the absence of any peak around 1400 cm^−1^ is evidence that this is phase pure^29^. Additional peaks at 840, 1300 and 1740 cm^−1^ can be attributed to the ITO^30^. The EDX spectrum shown in Fig. 4d also reveals the presence of peaks corresponds to iron, oxygen, silicon and contaminations from glass and suggest low impurity concentrations.

### Electrochemical Sensing of H_2_O_2_

**Figure 5:**
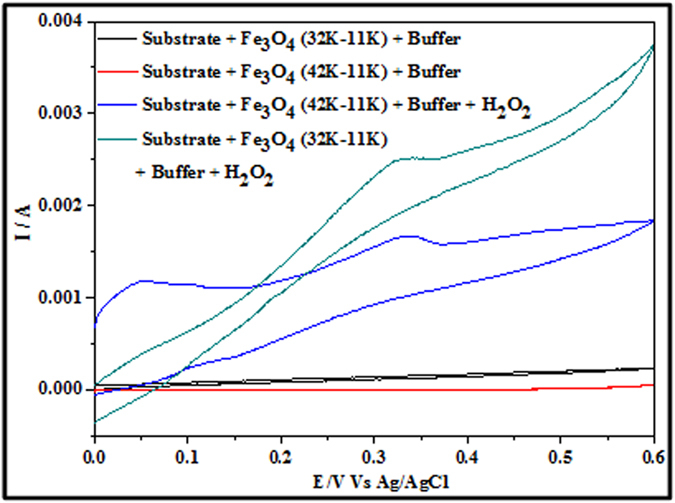
CV data showing the current response of sample ALW and sample BHW in the presence and absence of 2.5 μM H_2_O_2_ (phosphate buffer solution and scan rate = 50 mV s^−1^).

Fe_3_O_4_ nanodot – ITO samples were used as an electrochemical sensor for H_2_O_2_ detection. Typical CVs (scan rate = 50 mVs^−1^ in a 0.5 M phosphate buffer solution (pH = 7.4)) for ALW and BHW samples in presence and absence of 2.5 μM H_2_O_2_ are shown in Fig. 5. Both samples behave passively in this buffer solution and indicate the electrostability of the modified substrates. When H_2_O_2_ was introduced into modified electrode, a steep increase in anodic current was observed. An positive potential oxidation peak at 0.35 and 0.323 V was observed for sample BHW and ALW samples respectively and is associated with the oxidation of Fe(II) into Fe(III). The electrochemical reaction mechanism of Fe_3_O_4_ with H_2_O_2_ sensing and the intermediate by-products formation can be described by the reactions^31^:


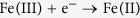



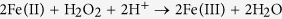


Here, Fe_3_O_4_ acts as the electrocatalyst responsible for H_2_O_2_ detection through the Fe(II)/Fe (III) redox couple. H_2_O_2_ oxidation potentials as low as 0.323V are rather low compared to reported literature values and suggests a real catalytic effect and the development of active sites at the nanodots^32^. The oxidation current of 2.7 mA for sample ALW is significantly larger than that for sample BHW (1.6 mA). This ratio of the anode currents (~1.7) are consistent with the coverage of nanodots that can be estimated at the surface using their known area and the surface density. For ALW and BHW samples the measured relative coverage are 0.28 and 0.19 respectively, a ratio of 1.55. Thanvadan *et al.*^33^ have observed similar H_2_O_2_ oxidation behaviour with an enzyme promoted nanoparticulate Fe_3_O_4_ system whereas in this study no enzyme and other treatments were used for H_2_O_2_ detection.

**Figure 6:**
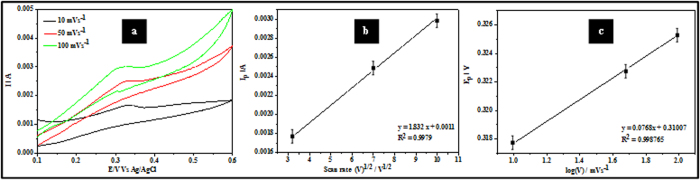
(**a**) CV data showing the current response of sample ALW (2.5 μM H_2_O_2,_ phosphate buffer solution) at various scan rates. (**b**) I_p_ vs 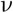
^1/2^ and (**c**) Tafel plot of Ep vs log 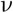 for the anodic process.

Figure 6a illustrates typical CV data from sample ALW recorded at scan rates of 10, 50 and 100 mVs^−1^ in the presence of H_2_O_2_. As might be expected, increasing scan rate results in sharper features and increased anodic peak currents from 1.7 mA to 3.0 mA. The proportionality of the peak current to the scan rate indicates a diffusion controlled electrochemical process. The diffusional charge transport is determined by ion transport or electron self-exchange in the Fe^3+^ and Fe^2+^ redox couple in Fe_3_O_4_. The motion of counter ions is required for electroneutrality and rapid electron transfer is generally favoured by the high redox site concentration^34^. The characteristics of the redox process can be ascertained from the Randles-Sevčik equation (Equation 1)^20^ by plotting peak current, I_p,_ against (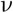
^1/2^) where 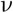 is the scan rate:


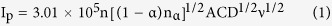


Here, n is the number of exchange electrons, α is the electron transfer coefficient, A is the electrode surface area, C is the concentration coefficient of the analyte, and D is the diffusion coefficient of the analyte. The linearity of the plot in Fig. 6b (regression coefficient, R^2^ = 0.9979) suggests a diffusion controlled redox process. The charge transfer coefficient (α) can be estimated from CV experiments using the Laviron method^35^ and plotting peak potentials against the logarithm of scan rate (Tafel plot). Figure 6c shows a linear variation of the anodic peak potential (E_p_) with log 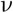 and described by E_p_ = 0.31007 + 0.0768 log 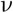. The slope of this plot is related to α and given by 2.303RT/(1 – α) nF and α can be estimated as 0.61. The Tafel plot can also be used to estimate the stability of the sample through a number of electro-oxidation processes using the Tafel equation (Equation 2) as given below^20^:


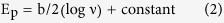


The value of b was estimated from the slope of the Tafel plot Fig. 6c and was found to be 153 mVdec^−1^.

**Figure 7:**
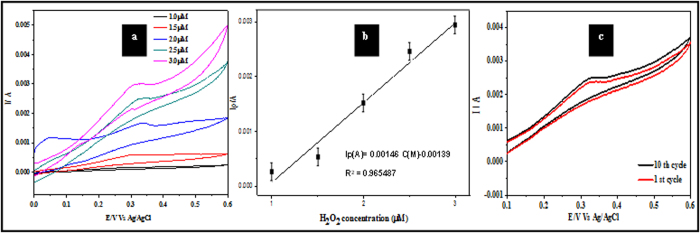
(**a**) CVs of sample ALW (phosphate buffer, scan rate 50 mV s^−1^) at different concentrations of H_2_O_2_. (**b**) Ip vs concentration of H_2_O_2_. (**c**) Summary of multiple scanning data (10 runs) of sample ALW in 2.5 μM H_2_O_2_.

Figure 7a illustrates the CVs for the ALW nanodots samples at increasing concentrations of H_2_O_2_ (1.0 μM to 3.0 μM, scan rates = 50 mVs^−1^). Figure 7b shows that the measured peak current is proportional to concentration (R^2^ = 0.991). This is consistent with an electrocatalytic mechanism as shown by Ojani *et al.*^36^). The detection limit, DL, can be estimated from the relationship DL = 3.3 s/m^20^, where s is the standard deviation of the intercept and m is the slope of the linear current *vs* H_2_O_2_ concentration. The estimated DL value is 3.96 μM. The sensitivity, S, of sample ALW as an electrode was calculated using S = SA(σI/σC) where σI/σC is the slope of Fig. 7b and SA is the sample surface area. S was estimated as 0.04 μA mM^−1^. Continuous multiple cyclic voltammetry scans of 50 cycles were performed for sample ALW in 2.5 μM H_2_O_2_ at a scan rate of 50 mV s^−1^ as shown in Fig. 7c. There were initially small changes from scan to scan but after 10 scans the data became experimentally indistinguishable and so stability is indicated by showing the first and 10^th^ scans only. The slight changes seen in the figure (a decrease of peak current of ~1.2%) might be due some poisoning of the surface by adsorption of impurities from the solution.

### Electrochemical Determination of EtOH Concentrations

**Figure 8:**
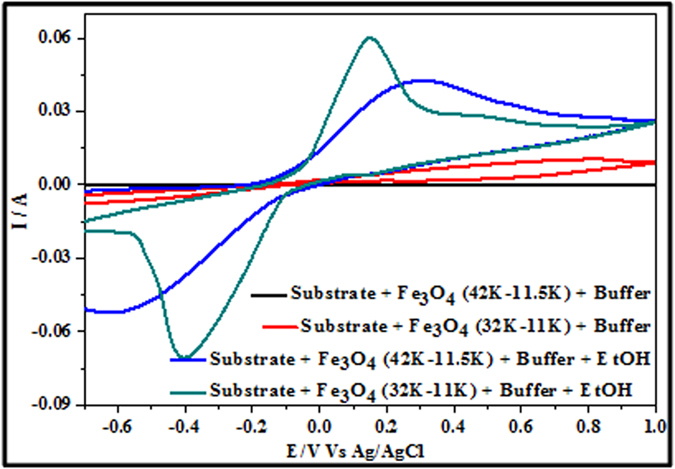
CV scans showing the current response of sample ALW and sample BHW in presence and absence of 0.1 M EtOH.

**Figure 9:**
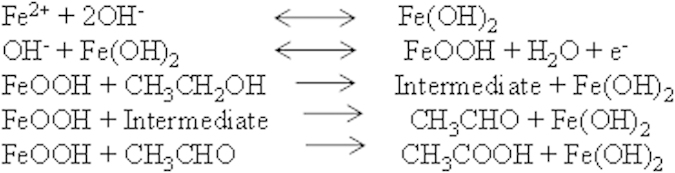
Ethanol oxidation mechanism at Fe_3_O_4_ electrode.

Comparison of the electrochemical behaviour of the nanodot samples from ALW and BHW to ethanol (normal buffer conditions as above and will not be described further) are shown in Fig. 8. Neither sample exhibited redox peak features at scan rate of 50 mV s^−1^ indicating passive behaviour. Addition of 0.1 M EtOH, resulted in well defined redox peaks at 0.16 and −0.4 V for sample ALW and 0.24 and −0.58 V for sample BHW. This smaller oxidation and reduction potentials of sample ALW compared to BHW confirm the enhanced electrocatalytic efficiency of the smaller nanodots. The reduction potential of about −0.4 V for ALW is lower than the literature values reflecting the small dimensions^21^. For sample ALW, the separation of cathodic and anodic peak potentials is ΔE = 0.24 V and the ratio of peak anodic and cathodic currents is I_pc_/I_pa_ = 1.02 V. This suggests that the electrochemical behaviour is quasi-reversible^37^. The enhanced current response (ratio ~ 1.45) and faster kinetics (peak width) of ALW compared to BHW reflect its higher surface coverage (coverage ratio = 1.55). For ethanol, the acid-base properties of the electrolyte can have a major influence on the observed oxidation potential^28^. During the electro oxidation process, intermediates species like CO, CO_2_, CH_3_CO, CH_3_CHO, CH_3_COO^−^, CH_3_COOH are formed and electrode poisoning can occur due to the re-adsorbed CO molecules. The mechanism of EtOH oxidation in weakly basic conditions can be summarised as shown in Fig. 9. The Fe^2+^ ions in Fe_3_O_4_ forms Fe(OH)_2_ and these are oxidized to Fe^3+^ in FeOOH. The FeOOH oxidizes ethanol forming reactive intermediates such as CH_3_CHO which are further oxidized to ethanol^37^,^38^. Since the oxidation rate of ethanal is rapid, this will be continuously oxidized to the acid. During the reverse sweep process, these carbonaceous elements may cause electrode poisoning due to adsorption of carbon species^39^. The reductive cycle enables the formed FeOOH to be reduced reactivating the surface of the electrode. Thus, it is the redox pair Fe(OH)_2_/FeOOH in the electrolyte medium leads to electrocatalytic activity towards EtOH sensing. In these CVs, the oxidation and reduction peaks represent the interconversion of the electrode surface from Fe(OH)_2_ to FeOOH. Note that the absence of an intense anodic oxidation peak at about −0.45 V indicative of the re-oxidation of EtOH and other carbonaceous species is consistent with the mild pH conditions as previous observations in strongly acidic or basic electrolyte medium^40^.

**Figure 10:**
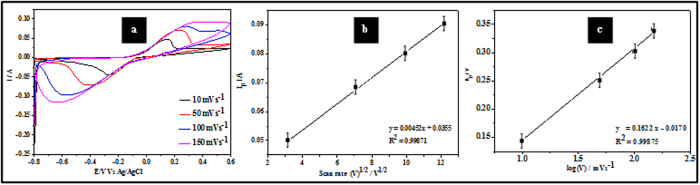
CVs showing the current response of sample ALW in 0.1 M EtOH at various scan rates. (**b**) I_p_ vs 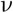
^1/2^ for anodic process. (**c**) Tafel plot of Ep vs log 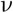 for the anodic process.

Figure 10a shows CV data of EtOH oxidation (sample ALW, 0.1 M EtOH) at various scan rates from 10 mV s^−1^ to 150 mV s^−1^. Figure 10b shows the plot of the anodic peak current I_p_ *vs* 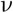
^1/2^ (see Equation (1)) and good linear dependence was observed (R^2^ = 0.9987). The reductive peak current behaved similarly. These data suggest that direct electron transfer between EtOH and the modified electrode surface occurs as shown in Fig. 10. Note that no intersection of anodic-cathodic current was observed for any of the scan rates used indicating that even at the lowest scan rate of 10 mV s^−1^ all of the EtOH is oxidized in the forward scan. The stability of sample ALW was estimated at 324 mV dec^−1^ using Equation 2 and the Tefal plot data in Fig. 10c as described above. Note that the value of b estimated is lower than seen previously^41^ which suggests that poisoning of the electrode is negligible during the reaction mechanism. This is important because it reflects the resistance of this oxide to degradation due to impurities. The calculated value for the anodic transfer coefficient (α) is 0.81 for EtOH oxidation process.

**Figure 11:**
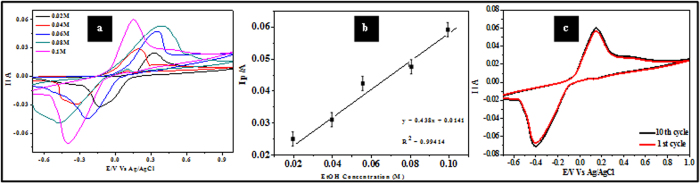
(**a**) CV data showing the current response of sample ALW at different concentrations of EtOH (scan rate = 50 mV s^−1^). (**b**) Ip vs concentration of EtOH. (**c**) Multiple scanning (10 runs) of sample ALW in 0.1 M EtOH in same conditions.

Figure 11a shows the CV of sample ALW modified electrode performances for varying EtOH concentrations of 0.02–1.0 M. The anodic peak current is linearly dependent on concentration (R^2^ = 0.9941) and varies from 25 to 60 mA (Fig. 11b). The detection limit was calculated as 5.52 mM and the sensitivity of sample ALW is 0.039 μA mM^−1^. A linear like current region was apparent for low EtOH concentrations which becomes sharper with increasing the concentration. This suggests that the mass transfer effect was eliminated for high EtOH concentrations (0.06–0.1 M) but it could not be eliminated for low EtOH concentrations (<0.04 M). Moreoever, at higher concentrations of EtOH ALW samples are very sensitive and so oxidation occurs even at lo potential than at lower concentrations. EtOH oxidation might be dominated by the kinetic effect (electron transfer) at high EtOH concentrations, and by mass transfer and electron transfer at low EtOH concentrations. Continuous CVs (50) of sample ALW in 0.1 M EtOH were preformed to test the long term use of these materials and are shown in Fig. 11c. As above, there was no measurable difference in data after 10 cycles and only the first and 10^th^ cycles are shown for clarity. Less than 1% variation in peak currents was observed.

### Dual Electrochemical Detection of H_2_O_2_ and EtOH by Nanodot Samples

**Figure 12:**
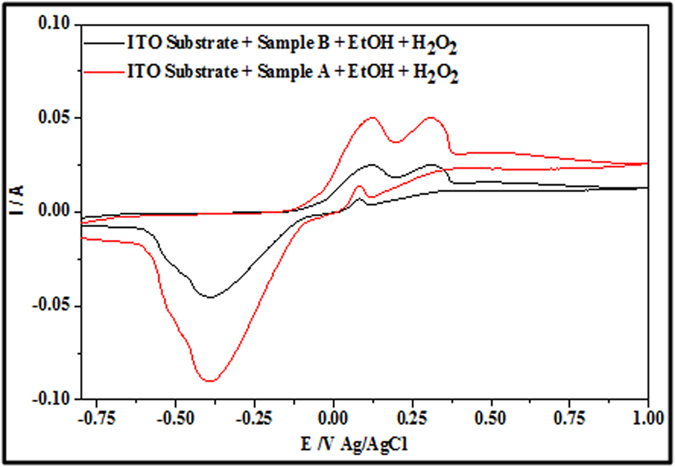
CV data showing electroxidation by Fe_3_O_4_ nanodots arrays for dual detection of EtOH and H_2_O_2_ at a scan rate of 50 mV s^−1^.

CVs in Fig. 12 provide evidence for the simultaneous detection of EtOH and H_2_O_2_ using sample ALW and sample BHW. Two distinct oxidation peaks appears for both samples at ~0.12 V and ~0.32 V consistent with EtOH and H_2_O_2_ oxidation. The data suggest that dual detection of these molecules is possible even at these low peak separations of ~0.2 V. It might be argued that the nanostructured form of the iron oxide in this system and the crystalline morphology/phase enables rapid oxidation and reduction and enabling this dual detection. Electrochemical dual detection of these two compounds is a challenge but demonstrated here. It should also be noted that extensive retesting over a period of 1 month indicated no significant loss of sensitivity and response and illustrated the stability of these systems.

## Discussion

Highly packed dot patterns of ALW and BHW are obtained by using a simple solvent annealing process. The vertical orientation depends on the annealing solvent which was achieved by toluene. Previously reported work suggests a PEO wetting layer on substrate surface due to high surface energy of PEO over PS. The annealing process depends on a combination of two major effects: (1) PEO has a higher surface tension (γ_C_ = 43 mNm^−1^) than PS (γ_C_ = 33 mNm^−1^) that would lead to preferential PS surface segregation; (2) this may be compensated by the fact that the annealing solvent is toluene, δ_Hildebrand_  ∫ δ_H_ = 18.2 (MPa)^½^, which is a preferential solvent for PS, δ_H_ = 18.7 (MPa)^½^, over PEO, δ_H_ = 20.3 (MPa)^½^. Thus PS observes more toluene than PEO leads to no wetting layer at the top of BCP film. The porous PS template of was obtained by a careful ultrasonication of ALW and BHW in ethanol for prescribed time. Longer exposure of ethanol vapour produces more structural degradation of the BCP template. This is avoided by optimising the exposure time for a set of increase in timings. A selective direct inclusion of iron oxide precursors in porous template was done by a simple spin casting followed by UV/ozone treatment. This oxidises the precursors into iron oxide nanodots and alternatively burns remaining polymers. This is further confirmed by XPS spectra. High resolution of Fe2p peak shows the crystalline phase of manufactures nanodots. The resultant feature size of iron oxide dots fabricated by ALW and BHW were 18 nm and 24 nm respectively. The samples of ALW and BHW were then used as electrochemical catalysts to sense ethanol and hydrogen peroxide.

Fe_3_O_4_ nanodots on ITO samples of ALW and BHW were initially subjected to electrochemical detection of H2O2 at a scan rate of 50 mVs^−1^. An oxidation potential and oxidation current of about 0.35 and 0.323 V and 1.6 mA and 2.7 mA were observed for BHW and ALW samples. The ratio of anode currents of ALW and BHW are ~1.7, which shows the higher density of nanodots on ALW rather than BHW. The increase in scan rate study of ALW samples from 50 to 100 mVs^−1^exhibits an increase in anodic currents from 1.7 mA to 3.0 mA. A constant increase in current with proportional to potential indicates diffusion enhanced electrochemical process. From the Tafel plot the stability of ALW samples for H_2_O_2_ detection was estimated as 153 mVdec^−1^. Further the increase in concentration of H_2_O_2_ from 1.0 μM to 3.0 μM shows the increase in anodic current peak. The detection limit and the sensitivity of ALW samples were calculated as 3.96 μM and 0.04 μA mM^−1^. Alternatively, ALW and BHW samples were undergone for ethanol detection and smaller redox potentials of sample ALW shows the better catalytic performance than BHW. The scan rate study of ethanol oxidation shows a linear increase in current and the stability of ALW samples towards ethanol oxidation was 324 mV dec-1. The anodic peak current varies from 25 mA to 60 mA when the concentration of EtOH was varied from 0.02 to1.0 M. The dual detection of EtOH and H_2_O_2_ using ALW and BHW samples shows two peaks at ~0.12 V and ~0.32 V. This proves that even at lower concentration of both EtOH and H_2_O_2_ can be detected by these Fe_3_O_4_ nanodots.

In summary, the BCP approach to creating the electrodes has been shown to be highly promising. It is apparent from the data that the nanodots prepared are both physically and chemically robust and electrodes can survive extended use without significant changes in detection capability or sensitivity. Iron oxide nanodots provided a useful platform because of the Fe^2+^/Fe^3+^ redox couple. The size and surface density of the nanodots produced electrodes of good electrocatalytic properties. The smaller molecular weight block copolymer films yielded better performing electrodes but this appeared to be largely related to higher surface coverage rather than any increase in active sites. Importantly, the work shows that with correctly nanostructured surfaces, efficient, robust non-enzyme based systems can be fabricated relatively easily. The avoidance of an enzyme promises much in terms of potential commercial opportunities since achieving long term stability of enzyme binding etc. is problematical. Further, these simple electrodes where extremely insensitive to impurity or self-contamination and could be used for very long periods without noticeable changes in either peak position or peak currents. This suggests that calibration of any commercial sensors would be limited.

This BCP mediated approach avoids many of the challenges of working with nanoparticulates such as attaching to a surface, aggregation (either in processing solution or during use e.g. by electromigration or sintering), powder handing/exposure concerns, size and thus property uniformity, nanoparticle synthesis etc. This approach can yield adhered nanodots of many materials in well-defined structural forms and arrangements. We would argue this shows that this chemistry can be exploited in a range of fields and applications. The sensitivity and detection limit for this linear range of concentrations for both H_2_O_2_ and EtOH is more efficient than previously reported works^33^,^35^,^39^,^42^.

## Methods

### Preparation of Iron Oxide Nanodots

Two different molecular weights of hexagonal forming polystyrene-*block*-poly(ethylene oxide) (PS-*b*-PEO) diblock copolymers, M_n_ = 32–11 kg mol^–1^, *M*_w_/*M*_n_ = 1.06; M_n_ = 42–11.5 kg mol^–1^, *M*_w_/*M*_n_ = 1.07; (where, *M*_n_ is the number-average molecular weight and *M*_w_ is the weight-average molecular weight) were purchased from Polymer Source Inc., Canada and used without further purification. These two PS-*b*-PEO systems subsequently are represented as ALW (lower molecular weight) and BHW (higher molecular weight) respectively. ITO coated glass substrates (with surface resistivity of 8–12 Ω per square and thickness of around 120–160 nm) were purchased from Sigma-Aldrich. These were cleaned by ultrasonication in ethanol and toluene for 30 min each and dried under a nitrogen stream. BCPs were dissolved in toluene to yield 1.0 wt% polymer solutions and were aged at room temperature for 12 h prior to film casting. PS-*b*-PEO thin films were spin coated onto the substrates at 3000 rpm for 30 s. The thin BCP films were solvent annealed by exposure to either toluene for 2 h (ALW) and toluene/water (50:50 v/v) mixed vapour for 1 h (BHW) under static vacuum at a temperature 50 °C to induce necessary chain mobility and allow microphase separation to occur (Fig. 1a). Partial etching (removal of PEO) and domain modification of PEO was carried out by ultrasonication of the films in anhydrous alcohol for different periods of time^22^ and this yields a nanoporous template for material inclusion (Fig. 1b). The films were then removed and dried immediately. To fabricate the iron oxide nanodots, different concentrations of iron (III) nitrate nonahydrate (Fe(NO_3_)_3_, 9H_2_O) were dissolved in ethanol and spin-coated onto the nanoporous films (Fig. 1c). UV/Ozone treatment was used to oxidize the precursor and remove polymer and so form the oxide nanodots as shown in Fig. 1d. All laboratory chemicals were of analytical grade (Sigma-Aldrich) and used as received unless otherwise stated. De-ionized (DI) water was used wherever necessary.

### Characterization

Surface morphologies were imaged by scanning probe microscopy (SPM, Park systems, XE-100) in tapping mode and scanning electron microscopy (SEM, FEI Company, FEG Quanta 6700). Transmission Electron Microscope (TEM) lamella specimens were prepared by a Zeiss Auriga-Focused Ion Beam (FIB) with a cobra ion column of 2.5 nm resolution and were analysed by an FEI Titan-TEM operating at an accelerating voltage of 200 kV. Compositional analysis was via energy dispersive analysis of x-ray (EDAX) and Raman spectroscopy. FTIR spectra were recorded on infrared spectrometer (IR 660, Varian). Raman spectra were recorded using a SPEX 1403 monochromator. The 488 nm line of an Ar ion laser was used for excitation at an output power of 20 mW. Film thicknesses were measured by optical ellipsometer (Woolam M2000) and electron microscopy. X-ray photoelectron spectroscopy (XPS) experiments were conducted on a Thermo K-alpha machine with Al K_α_ X-ray source operating at 72 W.

### Electrochemical Measurements

The electrochemical experiments were performed in the three electrode VersaSTAT 3 (Princeton Applied Research, USA) potentiostat tool that includes VersaStudio software. Ag/AgCl and KCl were used as reference electrodes and platinum wire as the counter electrode. Prior to experiments, solutions were de-aerated by bubbling nitrogen through them for 30 min at 293 K. The cyclic voltammetric (CV) curves for electro-oxidation of EtOH and H_2_O_2_ (30.0%) were measure in a 0.5 M phosphate buffer solution (pH = 7.4).

## Additional Information

**How to cite this article**: Rasappa, S. *et al.* A Highly Efficient Sensor Platform Using Simply Manufactured Nanodot Patterned Substrates. *Sci. Rep.* **5**, 13270; doi: 10.1038/srep13270 (2015).
